# SWISS-MODEL: modelling protein tertiary and quaternary structure using evolutionary information

**DOI:** 10.1093/nar/gku340

**Published:** 2014-04-29

**Authors:** Marco Biasini, Stefan Bienert, Andrew Waterhouse, Konstantin Arnold, Gabriel Studer, Tobias Schmidt, Florian Kiefer, Tiziano Gallo Cassarino, Martino Bertoni, Lorenza Bordoli, Torsten Schwede

**Affiliations:** 1 Biozentrum, University of Basel, Basel 4056, Switzerland; 2 SIB Swiss Institute of Bioinformatics, Basel 4056, Switzerland

## Abstract

Protein structure homology modelling has become a routine technique to generate 3D models for proteins when experimental structures are not available. Fully automated servers such as SWISS-MODEL with user-friendly web interfaces generate reliable models without the need for complex software packages or downloading large databases. Here, we describe the latest version of the SWISS-MODEL expert system for protein structure modelling. The SWISS-MODEL template library provides annotation of quaternary structure and essential ligands and co-factors to allow for building of complete structural models, including their oligomeric structure. The improved SWISS-MODEL pipeline makes extensive use of model quality estimation for selection of the most suitable templates and provides estimates of the expected accuracy of the resulting models. The accuracy of the models generated by SWISS-MODEL is continuously evaluated by the CAMEO system. The new web site allows users to interactively search for templates, cluster them by sequence similarity, structurally compare alternative templates and select the ones to be used for model building. In cases where multiple alternative template structures are available for a protein of interest, a user-guided template selection step allows building models in different functional states. SWISS-MODEL is available at http://swissmodel.expasy.org/.

## INTRODUCTION

SWISS-MODEL (http://swissmodel.expasy.org/) is an automated system for modelling the 3D structure of a protein from its amino acid sequence using homology modelling techniques. SWISS-MODEL has been established 20 years ago as the first fully automated server for protein structure homology modelling and has been continuously developed and improved since then (1–4). The server features a user-friendly web interface, which allows also non-specialists to generate 3D models for their protein of interests from a simple web-browser without the need to install and learn complex molecular modelling software or to download large databases (5). Today, SWISS-MODEL is one of the most widely used structure modelling web servers world-wide, with more than 0.9 million requests for protein models annually (i.e. ∼1 model per minute).

Recently, its functionality has been greatly extended: SWISS-MODEL now models oligomeric structures of target proteins and includes evolutionary conserved ligands such as essential cofactors or metal ions in the model. A newly developed interactive web interface allows users to conveniently search for suitable templates using sensitive Hidden Markov Models (HMM) searches against the SWISS-MODEL Template Library (SMTL), analyse alternative templates and alignments, perform structural superposition and comparison, explore ligands and cofactors in templates and compare the resulting models using mean force potential based model quality estimation tools.

Model quality estimation is an essential component of protein structure predictions, as the accuracy of a model determines its usefulness for practical applications. SWISS-MODEL provides model quality estimates (visually in the web page and numerically for download) based on a QMEAN potential (6,7) specifically re-parameterized for models built by SWISS-MODEL. The accuracy of the SWISS-MODEL server is independently evaluated in comparison with other state-of-the-art methods by the CAMEO project (http://cameo3d.org/; Continuous Automated Model EvaluatiOn) (8) based on target sequences weekly pre-released by the Protein Data Bank (PDB) (9).

## MATERIALS AND METHODS

### Overview

Homology modelling (or comparative modelling) relies on evolutionarily related structures (templates) to generate a structural model of a protein of interest (target). The process typically comprises the following steps: (i) template identification, (ii) template selection, (iii) model building and (iv) model quality estimation (10,11). In brief, a library of experimentally determined protein structures is searched with sensitive sequence search tools to identify proteins which are evolutionarily related to the target protein. If one or more templates are identified, the information of the alignment of the target and the template sequences together with the 3D coordinates of the template(s), are used to build a structural model for the protein of interest. Finally, the quality of the computed model is estimated to indicate the expected quality and suggest possible application of the obtained model.

### The SWISS-MODEL template library (SMTL)

Comparative modelling methods make use of information from experimentally determined protein structures to generate models for a target protein. A well-curated and annotated template library which supports efficient queries is therefore a crucial component of a modelling server. The SMTL aggregates information of experimental structures from the PDB (9) and augments it with derived information. When a new structure is released by the PDB, the coordinates and accompanying information are processed and imported into the template library. SMTL entries are organized by likely quaternary structure assemblies, termed 'bio units,' which are created according to the author- and software annotated oligomeric states listed in the PDB deposition. Template amino acid sequences are indexed in searchable databases for BLAST (12), and added to a HMM library that can be searched by HHblits (13). Sequence Profiles, predicted secondary structure (SSpro (14), PSIPRED (15)), predicted solvent accessibility (ACCpro (14)), per-residue solvent accessibility, (NACCESS (S. Hubbard and J. Thornton)), secondary structure (DSSP (16)) are calculated and stored alongside the structure. In addition, protein purification tags, such as poly-histidine or tandem affinity purification tags are detected in the sequences and marked as such. The implementation of computational routines in SMTL is based on OpenStructure (17).

### Annotation of ligands in SMTL

In most crystal structures low molecular weight ligands are observed, but only some of those are functionally or structurally relevant for the protein. Instead of their natural ligands, some structures contain synthetic analogues or inhibitors which occupy competitively the same binding site. Often, buffer or precipitant molecules are encountered, which are added by experimentalists to facilitate crystallization. SMTL implements a two-stage process to annotate biologically relevant ligands and synthetic analogues. The first stage uses a list of rules to automatically categorize the ligands based on their chemical identity. For example, all potassium ions are classified as solvent at this stage. In a second stage, the SMTL web interface provides a way to change the ligand classification manually. For example, in case of a potassium channel structure some of the before-mentioned potassium ions may be re-annotated as biologically relevant. While re-annotations can be suggested by any SWISS-MODEL user, before taking effect in SMTL, the annotations are reviewed by a curator to guarantee high quality of annotations.

### Template search and selection

The SWISS-MODEL Template Library is searched in parallel both with BLAST and HHblits to identify templates and to obtain target-template alignments. The combined usage of these two methods guarantees good alignments at high and low sequence identity levels (18). In order to select the most suitable templates, the procedure implemented in SWISS-MODEL uses properties of the target-template alignment (sequence identity, sequence similarity, HHblits score, agreement between predicted secondary structure of target and template, agreement between predicted solvent accessibility between target and template; all normalized by alignment length) to predict the expected quality of the resulting model (M. Biasini *et al.*, in preparation). In brief, each of the alignment properties is modelled as probability density function (PDF) of the estimate for a resulting model having a certain structural similarity to the target. The use of PDFs has the advantage of at once including the expectation value as well as the accuracy of the estimate for each property. It also takes into account, that some properties are better (more accurate) at predicting the quality at high levels of sequence identity, whereas others are more accurate in the twilight zone of sequence alignments. For each property the most likely structural similarity of the template to the target is the value at which the PDF is maximal. Properties are combined based on their relevance, which has been determined from large sets of target/template alignments with known target structures. When combining the estimates of each property, the most likely structural similarity is the value at which the joint distribution is maximized, termed the global quality estimation score (GMQE).

### Model building and scoring

After templates are selected for model building, either by using the automated or manual selection mode, the target/template alignment is used as input for generating an all-atom model for the target sequence using ProMod-II (19). In case loop modelling with ProMod-II does not give satisfactory results, an alternative model is built with MODELLER (10). By default, models are built using the homo-oligomeric structure of the template as annotated in SMTL, provided the oligomeric structure is predicted as conserved (see ‘Oligomeric structure prediction’ section).

An indispensable part of every modelling procedure is the estimation of a protein model's accuracy, directly providing the user with information regarding the range of its possible applications (11,20,21).

Here, model quality is assessed with the local composite scoring function QMEAN, which uses several statistical descriptors expressed as potentials of mean force: geometrical features of the model (pairwise atomic distances, torsion angles, solvent accessibility) are compared to statistical distributions obtained from experimental structures and scored. Each residue is assigned a reliability score between 0 and 1, describing the expected similarity to the native structure. Higher numbers indicate higher reliability of the residues. The weights of QMEAN have been specifically retrained for SWISS-MODEL, leading to more accurate local quality predictions for single models (G. Studer *et al.*, in preparation). In addition, global QMEAN scores are calculated as indicators for the overall model quality. Global QMEAN estimates are provided as a *Z*-score which relates the obtained values to scores calculated from a set of high-resolution X-ray structures (7). Additionally, a combined quality estimate is provided, which combines the QMEAN estimate with the GMQE obtained from the target-template alignment as described before. The resulting GMQE is again expressed as a number between zero and one, where higher numbers indicate higher reliability.

### Oligomeric structure prediction

The majority of proteins in a living cell exist as part of complexes and quaternary structure assemblies, monomeric proteins being the exception rather than the rule (22). Frequently, ligand binding sites and enzyme active sites are located at protein chain interfaces, and modelling of the oligomeric structure of a protein is therefore essential to build models which are useful in biomedical applications (23). Here, the homo-oligomeric structure of a target protein is modelled based on the hypothesis that the quaternary structure is conserved in one of the templates. To test this hypothesis, conservation of the oligomeric structure is predicted by analysing properties of interfaces between polypeptide chains such as sequence identity, sequence similarity, interface hydrophobicity and consensus occurrence of the same interface in the set of identified templates. A random forest is generated using these features as input parameters to predict the probability of conservation for each interface. When the size-weighted average of interface conservation is higher than a defined threshold, the oligomeric structure of the target is predicted to be the same as in the template.

### Modelling of ligands

For predicting essential ligands and cofactors for a given target protein, we apply a conservative homology transfer approach to small molecules which are observed in the templates identified in the SMTL. Ligands in SMTL are annotated either as: (i) relevant, non-covalently bound ligand, (ii) covalent modifications or (iii) non-functional binders (e.g. buffer or solvent). A non-covalently bound ligand is considered for the model if the coordinating residues are conserved in the target-template alignment. The relative coordinates of the ligand are transferred from the template, if the resulting atomic interactions in the model are within the expected range for van der Waals interactions and water mediated contacts.

### Performance of the method (CAMEO)

The performance and reliability of the SWISS-MODEL server is continuously evaluated by the CAMEO project (8). Modelling servers are blindly assessed based on sequences pre-released by the PDB for proteins whose structure will be published in the next release. Servers have 4 days to predict the 3D structure of the target proteins before models are evaluated against the protein structure coordinates released by the PDB using superposition-independent scoring methods such as Contact Area Difference (CAD) score (24) and local Distance Difference Test (lDDT) (25). The current CAMEO evaluation for this version of SWISS-MODEL consists of 6424 predictions for 599 target proteins collected over 52 weeks (i.e. from 1 March 2013 to 28 February 2014; data available at http://cameo3d.org). SWISS-MODEL accuracy is compared to other state-of-the-art protein structure prediction servers (26–32) and to previous version of the server (5).

### Webserver implementation

The web frontend to SWISS-MODEL follows the typical design of modern websites where business logic is implemented in JavaScript and executed directly in the browser. For improved user-interaction, data is fetched asynchronously from the server, without the need to reload the complete page. The front-end uses jQuery (jquery.com) to guarantee cross-browser compatibility. For 3D structure visualization, the user can chose between a modified version of OpenAstexViewer (openastexviewer.net) Java plugin, and the WebGL-based PV (https://biasmv.github.io/pv). The frontend communicates with a Django (www.djangoproject.com) backend that handles all incoming requests. Computationally demanding calculations, e.g. template search and modelling, are submitted via a queuing system to a dedicated compute cluster.

## SWISS-MODEL WEB INTERFACE

### Input

Model building with SWISS-MODEL can be initiated from different starting information: In the simplest case, a protein amino acid sequence can be specified directly (raw one letter sequence or FASTA format) or by referring to its UniProt accession code, in which case SWISS-MODEL will automatically retrieve the corresponding entry from UniProt (33). Alternatively, a target-template sequence alignment can be specified in the form of a multiple sequence alignment containing the target, the template and eventually other homologous sequences or in the form of a DeepView project file (3,19). At this point, the user can initiate the template selection step, which allows to manually select specific templates, or directly invoke the fully automated modelling pipeline.

### Output template search results and manual template selection

**Figure 1.**
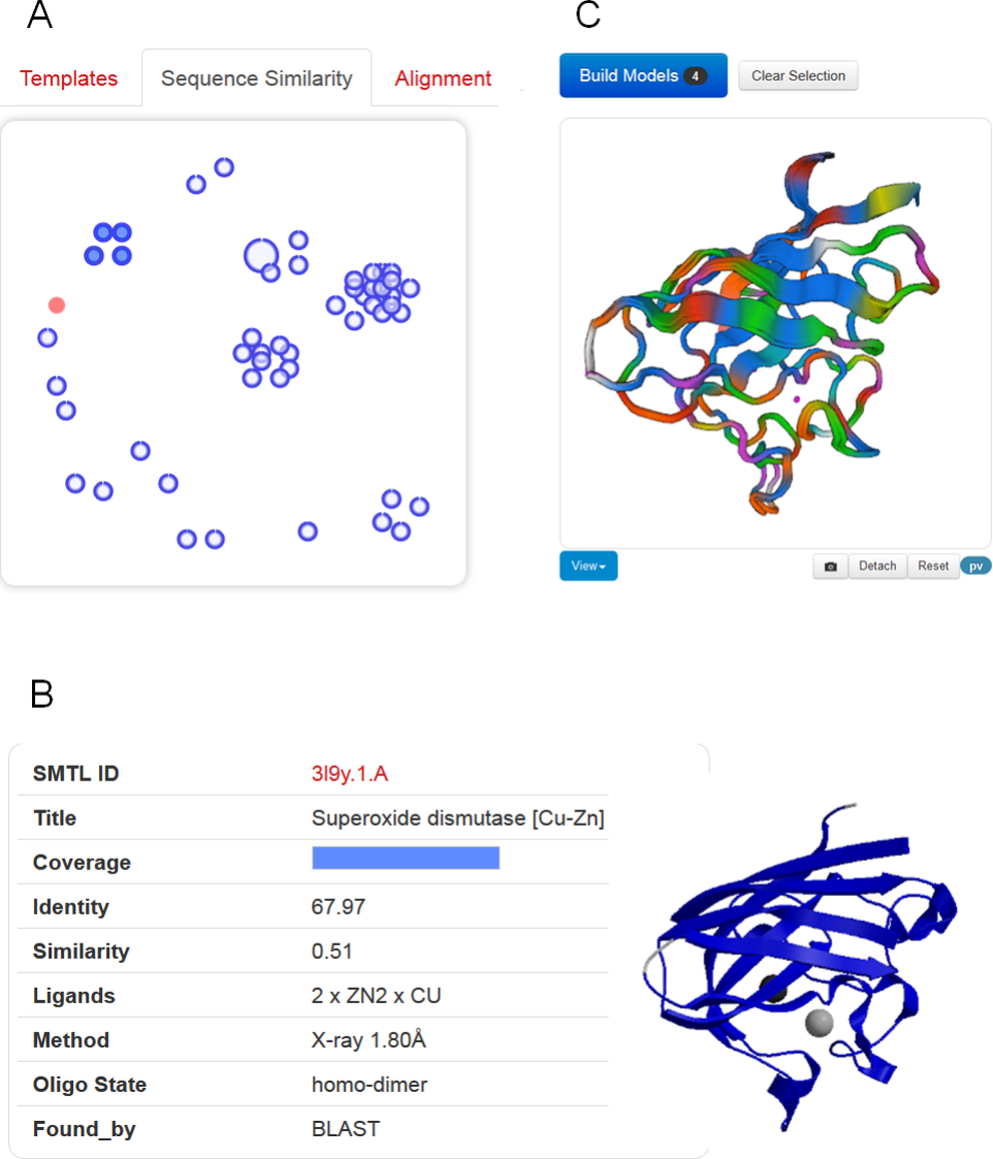
Templates selection and visualization. (**A**) An interactive chart shows the relationship of detected templates in sequence-similarity space. The target protein is represented as filled red circle. Each template is displayed as a blue circle, where the thick blue arc indicates target coverage (the N-terminus of the target protein starts at the top of the circle, and ends in clockwise direction with the C-terminus to close the circle). The distance between different templates is proportional to the pairwise sequence similarity, i.e. evolutionarily closely related templates will be clustered together. (**B**) Clicking on a circle will display template-specific information. A group of similar templates can be also visualized and selected by hovering over a cluster of templates. (**C**) The superposed structures of the selected templates will be instantaneously displayed in 3D to visually inspect structural differences.

Thanks to tremendous technical advances in experimental structure determination, for an increasing number of protein families there is not only one template, but multiple alternative template structures available. For some well-studied protein families, finding hundreds of possible templates for a target protein is not unusual. Often, these represent different functional states or structures in complex with different ligands. Depending on the intended application of a model, selecting a different template than the top-ranked one might be necessary, e.g. to build a model of a protein in complex with a ligand—rather than its apo form—for applications in drug design when induced fit movements are expected (34). We have therefore developed a manual template selection mode to make template selection available to a larger user base. All the steps of manual template selection can be performed directly in the web-interface without the need to leave the browser environment (Figure 1).

Suitable templates identified for the target sequence are listed in a tabular form, sorted by their predicted global quality estimation score (GMQE). Each template lists biologically relevant ligands, the predicted oligomeric structure conservation and the target-template alignment. The tabular view allows quickly gaining an overview on the identified templates. The user can directly select one or more templates and initiate model building. Apart from comparing template properties in tabular form, two graphical comparison views help to better understand the landscape of available templates. An interactive 3D view of superposed templates shows the aligned part of selected template structures (Figure 1C), at the user's choice using a WebGL-based (PV), or Java-based (OpenAstex) viewer. The second view shows the evolutionary distance between templates on 2D plot (Figure 1A). Groups of high-sequence identity templates cluster together, whereas more distant proteins are separated. The interactive graph allows marking groups of templates for structural superposition by selecting them with the cursor.

The sequence similarity cluster view in combination with template superposition allows identifying functionally relevant states of the templates (“open/closed”). It also supports defining structurally conserved cores in the identified template structures and such regions where templates which are not closely related share common structural features, are most likely well modelled in the target, while segments of structural variation in templates typically correlate with errors in the model (30,35).

### Output modelling

**Figure 2.**
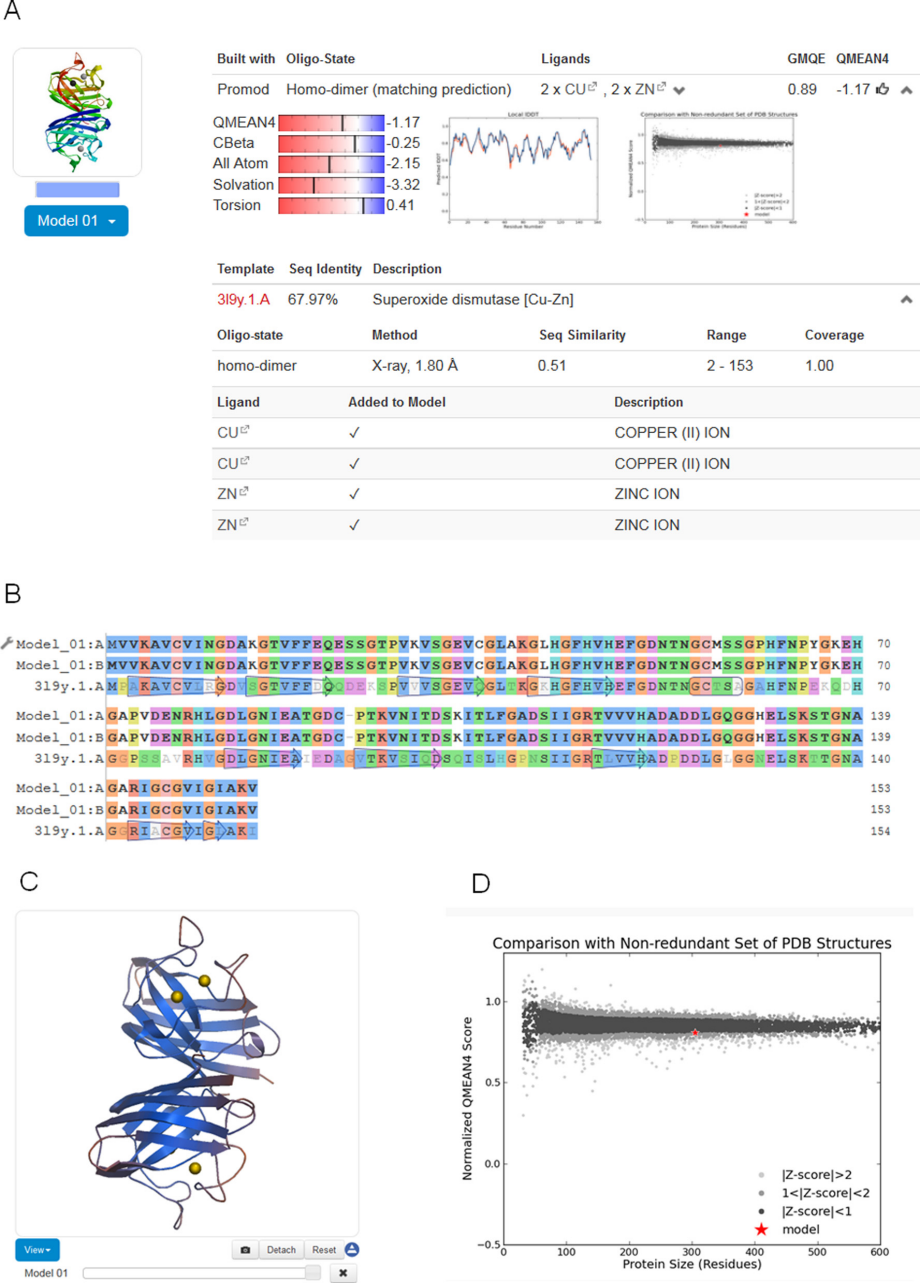
Modelling results. (**A**) For each model, coordinates, target-template alignment, modelling log and quality estimation information are provided. Information about the oligomeric structure, ligands and cofactors is also provided. (**B**) The colouring of the target-template sequence alignment can be changed to another scheme by clicking on the option button (adjustable spanner icon). Changes are simultaneously reflected in the structural representation of the model. (**C**) Models displayed in the interactive viewer are initially coloured by model quality estimates assigned by QMEAN. This allows instantly discriminating regions of the model which are well or poorly modelled. Local estimates of the model quality based on the QMEAN scoring function are shown as per-reside plot (A) and as global score in relation to a set of high-resolution PDB structures (*Z*-score) (**D**).

For each model generated based on the selected templates (either by the fully automated pipeline or interactively by the user), SWISS-MODEL provides the model coordinates along with relevant information to assess the modelling process and expected accuracy of the model (Figure 2): the target-template alignment, a step-by-step modelling log, information about the oligomeric state, ligands and cofactors in the model, as well as QMEAN model quality estimation. Models can be displayed interactively, initially coloured by model quality estimates assigned by QMEAN to highlight regions of the model which are well or poorly modelled. If several alternative models have been built for a target sequence, these can be interactively superposed and visualized. Model coordinates and information displayed on the website can be downloaded for later reference.

## CONCLUSION

Protein structure homology modelling has become a routine method to provide structural models on life science research in cases where no experimental structures are available. However, in order to support the understanding of a protein's function in its biological context, realistic structural models should not only correctly represent the overall fold of a single protein chain, but also its quaternary structure, as well as the atomic details of interactions with essential cofactors and ligands. Modelling and assessment procedures must also be able to account for structural flexibility since proteins are not static entities, but may exist in structurally distinct functional states.

With the new version of SWISS-MODEL presented here, we aimed to address these aspects by introducing a new augmented SWISS-MODEL Template Library, which includes information on quaternary structures and the role of ligands bound to the template. At the same time, we have significantly improved the accuracy of the fully automated SWISS-MODEL pipeline, aiming to reliably provide accurate models which are useful for applications in biomedical research. The expected accuracy of each specific model is communicated to the user in the form of QMEAN score, and the overall accuracy of SWISS-MODEL is continuously monitored in CAMEO. The implementation of the new web interface allows users to interactively compare alternative templates and select those which are more suitable for the intended application of the model (e.g. based on the presence/absence of specific ligands or structurally different functional states). The interactivity of the new web site required the usage of innovative programming techniques for the web front end, as well as speed optimization and hardware upgrades of the backend in order to provide a satisfying user experience.
